# Socio‐economic deprivation and functional exercise capacity: a retrospective observational cohort study

**DOI:** 10.1111/anae.70037

**Published:** 2025-10-10

**Authors:** Francesco Fiorini, George A. Rose, Damian M. Bailey, Richard G. Davies

**Affiliations:** ^1^ Cardiff University Cardiff UK; ^2^ Office of National Statistics Newport UK; ^3^ University of South Wales Pontypridd UK; ^4^ University Hospital of Wales Cardiff UK

Social determinants of health can influence surgical outcomes, with greater socio‐economic deprivation linked to lower survival following elective surgery [1] and emergency laparotomy [2]. Understanding the mechanisms underlying this inequality is a prerequisite for the design of targeted healthcare strategies to reduce its impact. Whether these differences stem from pre‐existing conditions, in‐hospital factors or events following hospital discharge remains uncertain.

Cardiopulmonary exercise testing (CPET) provides an objective measure of pre‐operative functional capacity, and variables are associated with postoperative complications and mortality [3]. While results vary by age, sex and comorbidity [4], their relationship with socio‐economic status remains poorly characterised [5]. We examined the potential association between area‐level deprivation, measured using the Welsh Index of Multiple Deprivation and CPET performance, in the largest peri‐operative cohort to date in Wales.

Ethical approval was granted by the University of South Wales and Cardiff and Vale University Health Board. All adults (aged ≥ 16 y) undergoing pre‐operative CPET at the University Hospital of Wales between 2008 and 2022 were included. Patients were not studied if testing was terminated for non‐physiological reasons or if they resided outside Wales. For multiple tests, only the most recent was used. The principal exposure was socio‐economic status, comprising income; employment; health; education; access to services; housing; community safety; and physical environment [6]. Quintiles were derived nationally, with Q1 representing the most, and Q5 the least, deprived areas. Patient, clinical and biochemical covariates were recorded. Exercise testing was performed using an electromagnetically braked cycle ergometer (Lode, Groningen, The Netherlands) and breath‐by‐breath analysis with a Medgraphics Ultima metabolic cart (MedGraphics™, Gloucester, UK), as described previously [7]. Primary outcomes were oxygen uptake at anaerobic threshold (V̇O_2_‐AT) and peak exercise (V̇O_2_ peak). Continuous variables were compared using the Kruskal–Wallis rank sum test and categorical variables using the χ^2^ test, where appropriate. Multivariable linear regression was used to model associations between Welsh Index of Multiple Deprivation quintile and oxygen uptake at V̇O_2_ peak and V̇O_2_‐AT, adjusting for relevant covariates.

**Figure 1:**
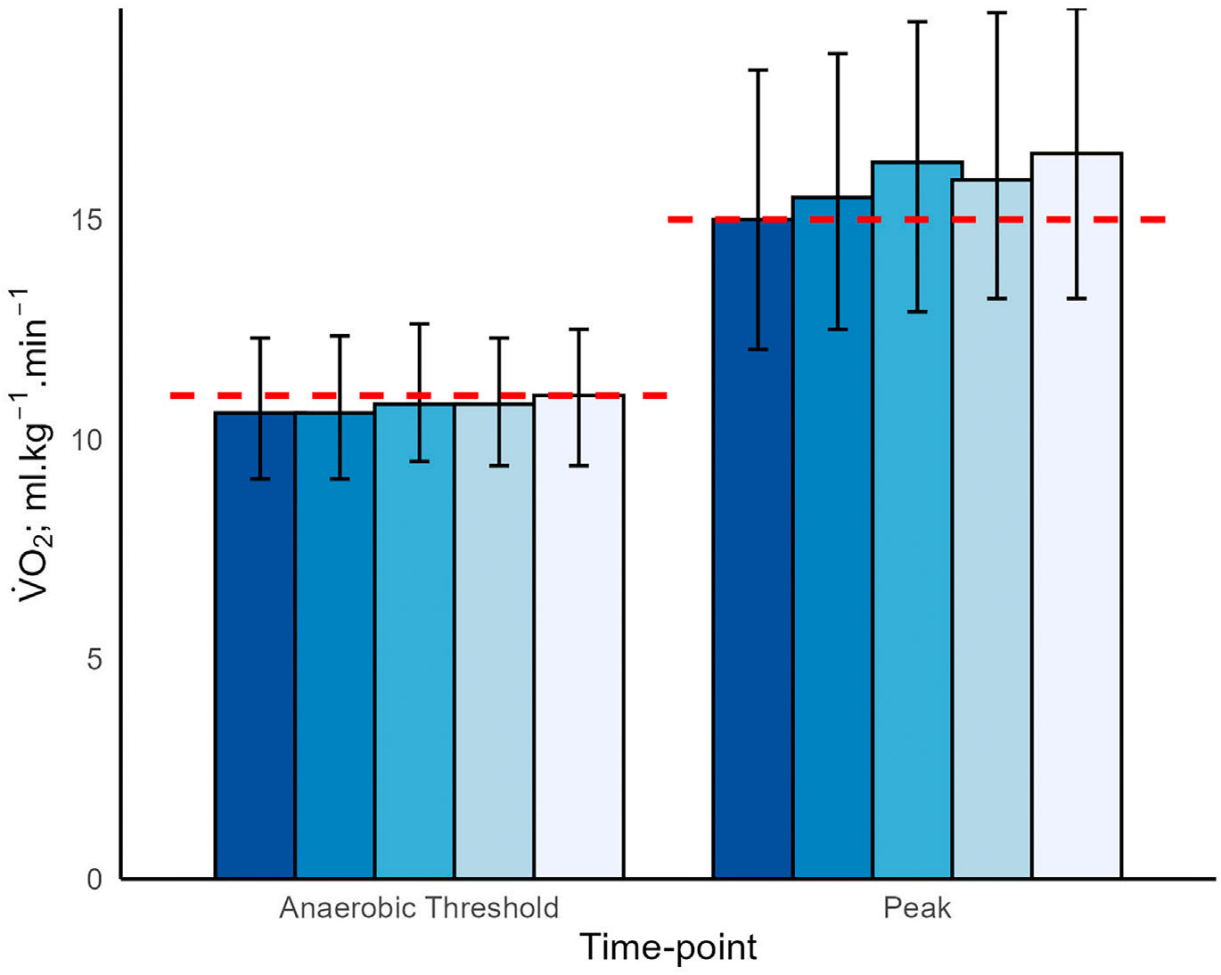
Median oxygen uptake (V̇O_2_) at anaerobic threshold and V̇O_2_ peak (error bars represent interquartile range), grouped by Welsh Index of Multiple Deprivation quintile from Q1 (dark blue, most deprived) to Q5 (pale blue, least deprived). The dashed red lines represent clinical risk thresholds commonly used for V̇O_2_ at anaerobic threshold and V̇O_2_ peak, respectively, 11 and 15 ml.kg^‐1^.min^‐1^.

A total of 3479 patients met inclusion criteria (Table 1). Patients in Q1 were younger, with higher BMI, comorbidity burden, smoking prevalence and reduced lung function compared with Q5 (p < 0.001). The median (IQR [range]) V̇O_2_‐AT was lowest in Q1 at 10.6 (9.1–12.3 [5.1–22.9]) ml.kg^‐1^.min^‐1^ vs. 11.0 (9.4–12.5 [5.2–26.0]) ml.kg^‐1^.min^‐1^ in Q5 (p = 0.015). Patients in Q1 reached V̇O_2_‐AT at lower metabolic equivalents (METs) than those in Q5. The median (IQR [range]) V̇O_2_ peak was lowest in Q1 at 15.0 (12.0–18.4 [5.9–32.2]) ml.kg^‐1^.min^‐1^ and greatest in Q5 at 16.5 (13.2–19.8 [5.2–38.6]) ml.kg^‐1^.min^‐1^ (p < 0.001). Patients in Q1 reached V̇O_2_ peak earlier than those in Q5 (541 vs. 594 s, p < 0.001), and at lower heart rate (125 beats.min^‐1^ vs. 130 beats.min^‐1^, p < 0.001), exercise workload (86 W vs. 92 W, p < 0.001), and METs (4.3 vs. 4.7, p < 0.001). There was a significant positive association between deprivation quintile and V̇O_2_ peak (β = 0.35 ml.kg^‐1^.min^‐1^, 95%CI 0.24–0.45, p < 0.001). This remained significant following adjustment for baseline characteristics (age, sex, BMI, smoking status and surgical specialty), clinical variables (comorbidities, ischaemic heart disease, chronic obstructive pulmonary disease, peripheral vascular disease and anaemia); baseline spirometry; medications (use of beta blockers, lipid‐lowering drugs); and for these combined (adjusted β = 0.21 ml.kg^‐1^.min^‐1^, 95%CI 0.13–0.29, p < 0.001). Baseline cardiopulmonary exercise testing measurements at rest showed no significant differences across deprivation quintiles, suggesting disparities are related to a dynamic response to exercise (Fig. 1).

**Table 1:** Baseline patient and clinical characteristics, including variables with prevalence >10% or significant variation across Welsh Index of Multiple Deprivation quintiles (p < 0.05). Values are mean (SD) or number (proportion).

	Welsh Index of Multiple Deprivation quintiles				
	Q1	Q2	Q3	Q4	Q5
	n = 802	n = 509	n = 514	n = 551	n = 1103
Age; y	67 (11)	67 (11)	67 (11)	68 (11)	71 (10)
Sex; female	271 (34%)	180 (35%)	176 (34%)	204 (37%)	404 (37%)
BMI; kg.m^‐2^	28.8 (6.3)	28.4 (6.1)	28.6 (5.9)	28.2 (5.6)	27.3 (4.9)
Current smoker	218 (27%)	137 (27%)	99 (19%)	80 (15%)	112 (10%)
Specialty					
Colorectal	406 (51%)	220 (43%)	241 (47%)	281 (51%)	720 (65%)
Upper gastrointestinal	163 (20%)	119 (23%)	112 (22%)	115 (21%)	161 (15%)
Hepatobiliary	97 (12%)	75 (15%)	85 (17%)	81 (15%)	101 (9%)
Other	136 (17%)	95 (19%)	76 (15%)	74 (13%)	121 (11%)
Comorbidity; ≥ 1	642 (80%)	399 (78%)	363 (71%)	410 (74%)	802 (73%)
Hypertension	367 (46%)	237 (47%)	223 (43%)	223 (40%)	478 (43%)
Coronary artery disease	122 (15%)	73 (14%)	47 (9%)	67 (12%)	116 (11%)
Myocardial infarction	72 (9%)	45 (9%)	41 (8%)	38 (7%)	61 (6%)
Cardiac failure	109 (14%)	67 (13%)	41 (8%)	48 (9%)	118 (11%)
Chronic obstructive pulmonary disease	126 (16%)	82 (16%)	53 (10%)	54 (10%)	70 (6%)
Asthma	90 (11%)	55 (11%)	47 (9%)	73 (13%)	99 (9%)
Diabetes	190 (24%)	100 (20%)	85 (17%)	103 (19%)	167 (15%)
Peripheral vascular disease	105 (13%)	68 (13%)	57 (11%)	53 (10%)	101 (9%)
Beta blocker use	190 (24%)	104 (20%)	120 (23%)	115 (21%)	230 (21%)
Lipid‐lowering drug use	392 (49%)	233 (46%)	210 (41%)	233 (42%)	460 (42%)
Haemoglobin; g.l^‐1^	131 (18)	131 (19)	133 (18)	133 (18)	131 (19)
Anaemia	287 (36%)	193 (38%)	185 (36%)	170 (31%)	410 (37%)
Albumin; g.l^‐1^	36.9 (4.6)	37.0 (4.7)	37.2 (4.6)	37.6 (4.4)	37.4 (4.3)

In this large peri‐operative cohort in Wales, socio‐economic deprivation was associated independently with reduced exercise capacity. These findings support previous work [8] and suggest that inequality exists even before hospitalisation, highlighting opportunities for targeted intervention. Tailored peri‐operative strategies, including prehabilitation and comorbidity optimisation, may help mitigate these disparities. Future integration with surgical outcome data will clarify their role in shaping postoperative risk.
